# Early Onset of Metastatic Gestational Trophoblastic Disease after Full-Term Pregnancy

**Published:** 2008-03

**Authors:** Fatemeh Ghaemmaghami, Mojgan Karimi Zarchi

**Affiliations:** 1 *Tehran University of Medical Sciences, Tehran, Iran;*; 2 *Department of Gynecologic oncology, Vali-e-Asr Hospital, Imam Khomeini Oncology Complex, Tehran, Iran*

**Keywords:** GTD, Choriocarcinoma, vaginal bleeding after delivery, EMA/CO

## Abstract

Choriocarcinoma is a curable malignancy that occurred approximately 50% after term pregnancies, and prognosis in this form of gestational trophoblastic Disease (GTD) is Poor. The earliest onset choriocarcinoma after term pregnancy in one study was reported 3 weeks after delivery, but in current study, choriocarcinoma was diagnosed 2 weeks after delivery. 28 years-old women gravidity 2, parity 2 delivered a healthy infant at term. Frequent episodes of vaginal bleeding occurred after 10 days of delivery. On admission to hospital, she had lesions in the lungs. The pretreatment human chorionic gonadotropin (HCG) level was 84,000 mIU/ml and her FIGO risk factor score was 8 (high risk group). The EMA/CO regimen was administered as first line chemotherapy and the patient achieved complete remission after 7 courses. Although early onset postpartum hemorrhage is due to complication of delivery, but gestational trophoblastic disease (GTD) may be occurred and assessment of human chorionic gonadotropin could be help to early diagnose of GTD.

## INTRODUCTION

Gestational Trophoblastic Disease (GTD) represents a variety of conditions that include hydatiform mole, invasive mole, choriocarcinoma and Placental Site Trophoblastic Tumor (PSTT). Choriocarcinoma is a rare trophoblastic tumor, which was curable and may be developing after any gestational events. Choriocarcinoma occurred approximately 50% after term pregnancies, 25% after molar pregnancies and the reminder after other gestational events (1-3). Choriocarcinoma follows a normal term pregnancy in 1 per 150000-160000 normal pregnancies and it is associated with an unfavorable outcome. GTD after a normal pregnancy is always choriocarcinoma. (1-4) Choriocarcinoma invades and metastasis early and is often widespread at the time of diagnosis. Presently signs and symptoms of gestational choiocarcinoma are highly variable in patients with GTD, gynecological symptoms are sometime ignored, attributed to normal peripartum or vaginal bleeding in puerperium or may be presented by non classic manifestation (1-3). Multiple studies have been reported about choriocarcinoma after term pregnancy (Table 1). Berkowitz reported that incidence of choriocarcinoma after term delivery was 4.1% of 366 case of GTD. Post term gestation choriocarcinoma has a propensity for more extensive metastatic spread particular liver and brain and remission rate in patients to conventional chemotherapy was lower than other forms of GTD (Remission rate=61.5%) (3). Miller has reported that poor prognostic factors in choriocarcinoma after term delivery included:

- Initial human chorionic gonadotropin titer of greater than 100,000 IU/24 h;
- Interval >4 month between termination of pregnancy to initial treatment;
- Previous failure chemotherapy;
- Brain or liver metastasis.

**Table 1:** Case report of post term delivery choriocarcinoma

Authors	Interval from index pregnancy	Sign & Symptom	Site of metastasis	Treatment: Chemotherapy

Farely [2005] (4)	6 months	AUB & rise of BHCG	Lung metastasis	Oral MTX
Flam [1996] (7)	11 weeks	ResistanceVaginal bleeding with normal placenta	?	?
Yuji [2005] (8)	3 weeks	Headache & hemoptisia	Lungs & brain metastasis	EMA/CO regimen
Current study [2007]	2 weeks	Resistance vaginal bleeding & rise of BHCG	Lungs metastasis	EMA/CO regimen

He also reported that response rate in choriocarcinoma after term delivery to be less than other forms of GTD (4). Also if interval from index pregnancy to initial treatment to be less than 4 month, remission rate was 87.5%. He added, in post-term metastatic choriocarcinoma should be initiated combination chemotherapy and in special cases, surgery or radiotherapy was benefit. Lurain (5) had reported that remission rate in metastatic choriocarcinoma after term delivery was 50% VS 75% in other forms of GTD. Number of risk factors is important, for example: if only one or two risk factor is available, survival rate was 74%, but if 3 or 4 risk factor is available, this rate was 27%.

## CASE

**Figure 1:**
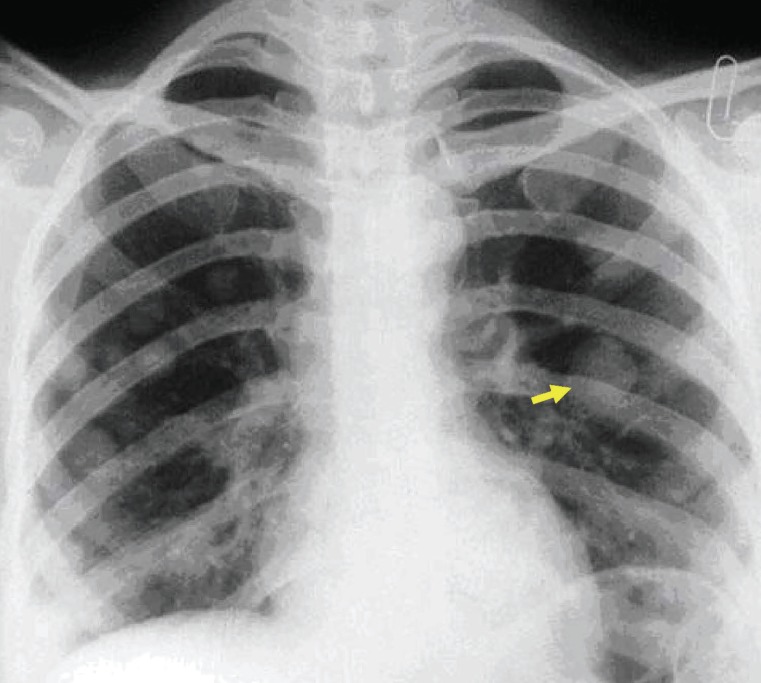
The appearance of lung metastasis in patient that was reported.

**Figure 2:**
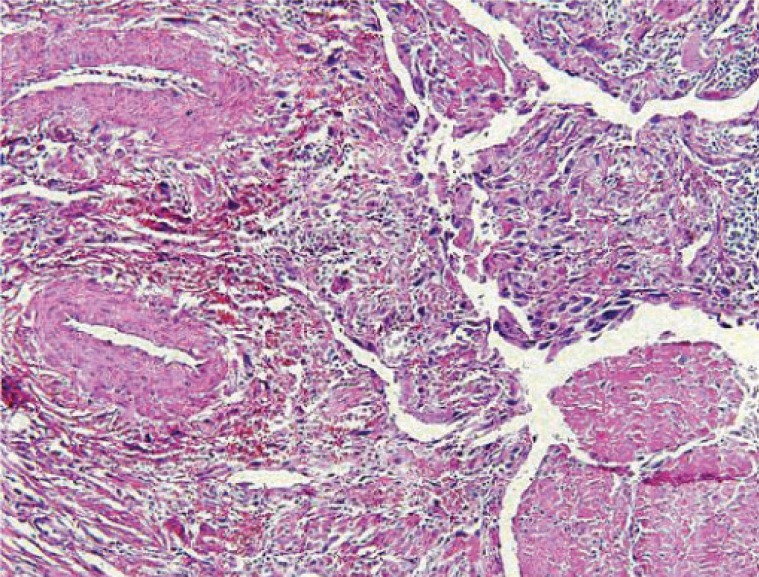
The microscopic appearance of choriocarcinoma in this patient.

A 28-years-old woman (gravida 2, parity2) who had delivered 3,200 g female infant on October, 2005. The placenta was macroscopically normal. The patient had vaginal bleeding greatrer than normal patients 10 days after delivery. Laboratory evaluation was a titer of 55,000 miu/ml in HCG. Pelvic ultrasound examination revealed a 12×9×8 cm uterus with a thick endometrial line. Due to high level of HCG titer & persistent bleeding, curettage was performed 2 weeks after delivery that established degenerative tissue. But her bleeding had increased after D&C and HCG had raised (to 84,000 miu/ml). In her chest radiography had diagnosed metastatic nodules (Fig. 1) and in lung CT scan revealed multiple bilateral pulmonary nodules. Due to sever bleeding, she was underwent total abdominal hysterectomy in 7 weeks after delivery for life saving and her pathology was choriocarcinoma (Fig. 2). The titrage of HCG after surgery was 83,000 miu/ml. According to the FIGO scoring system by the WHO, the patient score was 8 (high risk group) and her stage was III, then she was underwent combination chemotherapy with EMA/CO regimens (Etoposide+Metotrexate (MTX)+Actinomycin-d+cyclophosphamid+vincristin). At this time she had received 5 course of EMA/CO regimen and 2 courses for consalidation therapy were performed after complete remission currently. HCG titer decreased and was negative after 3 courses and the patient received 2 courses after normal range of HCG titer.

**Table 2:** EMA/CO regimen

**Course 1(EMA)**	
**Day 1**	Etoposide:100 mg/m^2^, i.v. Infusion in 200 ml of saline over 30 min
	Actinomycin D: 0.5 mg, i.v. push
	Methotrexate:100 mg, i.v. push followed by a 200 mg/m^2^, i.v., infusion over 12h
**Day 2**	Etoposide: 100 mg/m^2^, i.v. infusion in 200 ml of saline over 30 min
	Actinomycin D: 0.5 mg i.v. push
	Folic acid: 15 mg, i.v. or orally every 12 h for 4 days beginning 24 h after start

**Course 2 (CO)**	
**Day 8**	Vincristin: 1.0 mg/m^2^, i.v. push
	Cytoxan: 600 mg/m^2^, i.v. in saline

This regimen consists of 2 courses: 1) course 1 is given in days 1 and 2; 2) course 2 is given on day 8. These courses can usually be given on day 1 and 2, 8, 15 and 16, 22 etc., and the intervals should not be extended without course.

## DISCUSSION

Although the most common cause of post partum hemorrhage is complication of delivery, but should be remember that GTD may be occurred. In previous studies early onset metastatic choriocarcinoma after term pregnancy was 6 months, 11 weeks and 3 weeks, but in current study GTD after 2 weeks of delivery was diagnosed. Also all patients in these reports had lung metastasis (7-9). Prognosis of metastatic choriocarcinoma after term pregnancy is generally poor. Due to early extensive spread of disease and responsiveness to chemotherapy and change in the host immune response or a delayed diagnosis (4-6). The patients with metastatic choriocarcinoma shouldn’t be underwent biopsy of metastasis, due to increased risk of hemorrhage of metastasis (2-3). The other forms of GTD after term pregnancy is Placental Site Trophoblastic Tumor (PSTT) that has occurred approximately in 53-78% after term pregnancy. Vaginal bleeding or amenorrhea has seen in always of patients with PSTT. The different diagnosis of post-term choriocarcinoma and PSTT is level of BHCG, because in PSTT measure of HCG is lower than choriocarcinoma (9). In many of studies interval of disappearance of HCG after delivery is variable and mean of this time is 3 weeks (10). Remission rate in metastatic post-term gestational choriocarcinoma is lower than this after other forms of pregnancy (7-10). A Many of combination chemotherapy regimens had treated in choriocarcinoma, that includes: EMA/CO regimens include Etoposide+Metotrexate (MTX)+Actinomycin-d+cyclophosphamid+vincristin. Cure rate with MAC regimen in first line treatment was 51% VS 30% in second line treatment. Complete remission was seen with CHAMOCA regimen in patients that resistance to MAC, but this regimen is one protocol with high toxicity (5). In previous studies was shown that etoposide not only has effective in first line treatment, but also in recurrence of GTD (12). Also taxans has shown that has effective in recurrence cases. Without lower limit of tumoral cells that has diagnosed one titer of HCG for early diagnosed, is 10000 cells, thus some of authors had relieved that should be Continued 3 course of chemotherapy as consolidation therapy and some of they had commented that treatment should be continued to one negative titrage of HCG. Importantly, recurrence may be had seen many age after complete remission of GTN. Overall recurrence rate with EMA/CO: approximately 11-19% had reported (2). Well defined that response rate to secondary chemotherapy is poor, thus has recommended early treatment has selected well (9-12). We have thought that EMA/CO regimen should be selected as first line in always of patients with high risk GTD, because response rate is good and tolerance is Sufficient.
